# Tissue magnetic susceptibility mapping as a marker of tau pathology in Alzheimer's disease

**DOI:** 10.1016/j.neuroimage.2017.08.003

**Published:** 2017-10-01

**Authors:** J. O'Callaghan, H. Holmes, N. Powell, J.A. Wells, O. Ismail, I.F. Harrison, B. Siow, R. Johnson, Z. Ahmed, A. Fisher, S. Meftah, M.J. O'Neill, T.K. Murray, E.C. Collins, K. Shmueli, M.F. Lythgoe

**Affiliations:** a UCL Centre for Advanced Biomedical Imaging, Division of Medicine, UCL, UK; b Eli Lilly and Company, 355 E Merrill Street, Dock 48, Indianapolis, IN, 46225, USA; c Eli Lilly & Co. Ltd, Erl Wood Manor, Windlesham, Surrey, GU20 6PH, UK; d Department of Medical Physics and Biomedical Engineering, UCL, UK

## Abstract

Alzheimer's disease is connected to a number of other neurodegenerative conditions, known collectively as ‘tauopathies’, by the presence of aggregated tau protein in the brain. Neuroinflammation and oxidative stress in AD are associated with tau pathology and both the breakdown of axonal sheaths in white matter tracts and excess iron accumulation grey matter brain regions. Despite the identification of myelin and iron concentration as major sources of contrast in quantitative susceptibility maps of the brain, the sensitivity of this technique to tau pathology has yet to be explored. In this study, we perform Quantitative Susceptibility Mapping (QSM) and T2* mapping in the rTg4510, a mouse model of tauopathy, both *in vivo* and *ex vivo*. Significant correlations were observed between histological measures of myelin content and both mean regional magnetic susceptibility and T2* values. These results suggest that magnetic susceptibility is sensitive to tissue myelin concentrations across different regions of the brain. Differences in magnetic susceptibility were detected in the corpus callosum, striatum, hippocampus and thalamus of the rTg4510 mice relative to wild type controls. The concentration of neurofibrillary tangles was found to be low to intermediate in these brain regions indicating that QSM may be a useful biomarker for early stage detection of tau pathology in neurodegenerative diseases.

## Highlights

• The rTg4510 is a mouse model of tauopathy.
• We applied QSM and T2* Mapping MRI techniques to the rTg4510 *in vivo* and *ex vivo.*
• QSM demonstrated sensitivity to regions of low and intermediate tau burden.
• QSM may hold potential as a non-invasive early biomarker of tau pathology.

## 1 Introduction

Alzheimer's disease (AD) is defined by the presence of amyloid-β plaque and neurofibrillary tangle (NFT) tau pathology found primarily in grey matter regions of the brain. These insoluble plaques and tangles have both been found to contain iron (Lovell et al., 1998, Good et al., 1992). Iron levels need to be tightly regulated in the brain but homeostasis can become disturbed during neuroinflammation which is thought to increase iron levels in neurons and microglia (Urrutia et al., 2013). Oxidative stress is associated with the dysfunction of oligodendrocytes in AD (Cai and Xiao, 2015) and white matter degradation has been detected by histopathological examination in over 50% of patients (Sjöbeck et al., 2005). Numerous white matter changes in AD have been reported in post mortem studies including decreased myelin density (Sjöbeck et al., 2005), decreased myelin basic protein (Wang et al., 2004), loss of oligodendrocytes (Sjöbeck et al., 2006), activation of microglia (Gouw et al., 2008), as well as denudation of the ventricular ependyma, gliosis and the loss of myelinated axons (Scheltens et al., 1995). *In-vivo* biomarkers sensitive to tissue neuroinflammatory processes and the concentration of iron and myelin in brain tissue, may play a key role in tracking the progressive pathology of AD and provide a means by which to measure the efficacy of therapeutics.

Quantitative Susceptibility Mapping (QSM) (Wang and Liu, 2015, Shmueli et al., 2009, Haacke et al., 2015, Liu et al., 2014, Deistung et al., 2016), uses the phase of the MRI signal to calculate maps of the bulk magnetic susceptibility of tissue. Myelin is diamagnetic and has been shown to be a predominant source of susceptibility contrast between white and grey matter (Liu et al., 2011a, Klohs et al., 2013, Lee et al., 2012). Furthermore, magnetic susceptibility measurements in white matter regions using QSM have been shown to be more specifically related to myelin concentration than measures of diffusion using DTI (Argyridis et al., 2014). In addition to its dependence on myelin, magnetic susceptibility has been shown to correlate with iron concentrations in tissue (Langkammer et al., 2012, Bilgic et al., 2012), and, like findings of reduced T2*in AD (Moon et al., 2012, Zhao et al., 2017), these increases have been attributed to increased iron deposition.

In a recent clinical study, significant increases in magnetic susceptibility were detected in AD patients relative to controls in the putamen, a sub region of the striatum (Acosta-Cabronero et al., 2013). In the ArcAβ, an amyloid mouse model of AD, smaller susceptibility increases over time were observed relative to controls in a longitudinal study using a linear mixed effects modelling analysis that incorporated estimates from multiple brain regions (Klohs et al., 2013). Thus far, no susceptibility mapping studies have been performed in mice exhibiting tau pathology associated with AD.

The rTg4510 mouse model of tauopathy contains the P301L human tau mutation, and accumulates NFTs in a progressive manner along with motor and behavioural deficits that are similar to those in human AD (Lewis et al., 2000). Previous work in this model has identified abnormalities in the white matter of the corpus callosum using DTI and Electron Microscopy (Wells et al., 2015, Sahara et al., 2014). Additionally, reactive microglia and astrocytes, associated with neuroinflammation and iron accumulation, are known to be present in higher quantities in the rTg4510 than in controls (Maphis et al., 2015, Yoshiyama et al., 2007). We hypothesized that QSM might provide a sensitive *in-vivo* method to non-invasively probe these pathological traits of the rTg4510 mouse model.

In this study, we present *in-vivo* QSM and T2* maps in the rTg4510 mouse, supported by higher resolution measurements from *ex-vivo* datasets. A semi-automatic segmentation of the quantitative parameter maps was employed to calculate magnetic susceptibility and T2*values in selected grey matter and white matter regions. The biological factors contributing to magnetic susceptibility and T2* measurements in the tissue were investigated by comparison with histological stains for myelin, iron, and neuroinflammatory markers to aid interpretation of the MRI findings.

## 2 Materials and methods

### 2.1 Animals

Female rTg4510 transgenic mice were licensed from the Mayo Clinic (Jacksonville Florida, USA) and bred for Eli Lilly by Taconic (Germantown, USA) (Ramsden et al., 2005a). Mice were imported to the UK for imaging studies at the Centre for Advanced Biomedical Imaging, University College London. All studies were carried out in accordance with the United Kingdom Animals (Scientific Procedures) act of 1986.

### 2.2 In-vivo data acquisition

*In-vivo* imaging was conducted on rTg4510 mice (n = 10) and wild-type (WT) controls (n = 10) aged 7.5 months. Data were acquired with a 9.4 T VNMRS horizontal bore scanner (Agilent Inc.). A 72 mm inner diameter volume coil (Rapid Biomedical) was used for RF transmission and signal was received using a two-channel head array (Rapid Biomedical). Mice were anaesthetised under 2% isoflurane in 100% O_2_ and were immobilised by securing the head with a bite bar and ear bars. The anaesthesia was subsequently reduced to 1.5% isoflurane and maintained at this level throughout imaging. Core temperature and respiration were monitored using a rectal probe and pressure pad (SA instruments). Mice were maintained at ∼37 °C using heated water tubing and a warm air blower with a feedback system (SA instruments). Shimming was performed using an automatic 3D gradient echo shim function (VNMRJ, Agilent Inc.) in a voxel (1 cm^3^) centred in the cortex resulting in linewidths of 47 ± 7 Hz. Data for QSM was collected using a 3D single echo spoiled gradient recalled echo (GRE) sequence with first order flow compensation applied in three dimensions. Subsequently, a multi-echo sequence was run without flow compensation to acquire data for T2* mapping. Parameters for MRI pulse sequences are provided in Table 1. Flow compensation can improve phase estimation *in vivo* by reducing errors caused by mislocalisation of signal and accumulation of spins due to motion at a constant velocity (Haacke et al., 2015, Xu et al., 2014, Deistung et al., 2009). Consequently, the multi-echo data, which were not flow compensated, were not used for QSM.

**Table 1:** Imaging pulse sequences and parameters. For *in-vivo* acquisition, a flow compensated 3D GRE sequence was used to generate data for QSM and a Multi-Echo 3D GRE sequence for T2* mapping data. A Multi-Echo 3D GRE acquisition was used to collect *ex-vivo* QSM and T2* mapping data. Abbreviations: NE: Number of echoes, NSA: Number of signal averages.

Parameter ∖ Pulse seq.	*In-vivo* (QSM): Flow compensated 3D GRE	*In-vivo* (T2*): 3D multi-echo GRE	*Ex-vivo* (QSM/T2*): 3D multi-echo GRE
TR (ms)	250	250	200
minTE/ΔTE/maxTE (ms)	15	2.31/2.46/29.32	3.05/3.92/46.21
FA (^0^)	32	32	36
NE	1	12	12
NSA	1	1	5
Scan time	1hr,2s	1hr,2s	10hr,45min,2s
Spectral width (Hz)	50000	100000	73529
FOV (mm)	18 × 18 × 18	18 × 18 × 18	18 × 17.2 × 14.4
Matrix	120 × 120 × 120	120 × 120 × 120	225 × 215 × 180
Resolution (μm)	150 × 150 × 150	150 × 150 × 150	80 × 80 × 80

### 2.3 Preparation of ex-vivo samples

Animals were terminally anaesthetised with Euthanal administered via intraperitoneal injection immediately after *in-vivo* imaging. Fixation was then carried out by perfusion through the left ventricle: first with 15–20 mL of saline (0.9%) and heparin; second with 50 mL of buffered formal saline (10% solution, Sigma-Aldrich), at a flow rate of 3 mL per minute. Brains (in-skull) were then removed and stored at 4 °C in buffered formal saline. After 4 weeks, brains were transferred to phosphate buffered saline (50 ml PBS refreshed weekly, Sigma-Aldrich) for rehydration (Zhang et al., 2012, Shepherd et al., 2009, Benveniste and Blackband, 2002) for a further 3 weeks.

### 2.4 Ex-vivo data acquisition

*Ex-vivo* imaging was conducted on rTg4510 mice (n = 8) and wild-type controls (n = 8) using a 3D spoiled GRE acquisition with parameters given in Table 1. Four mice from the *in-vivo* cohort were excluded from *ex-vivo* processing due to non-optimal perfuse fixation. Each brain (in-skull) was secured individually in a 20 ml syringe filled with 10 ml proton MR signal-free, non-viscous Fomblin perfluoropolyether (PFS-1, Solvay Solexis SpA., Bollate, Italy) prior to imaging in a 26 mm diameter birdcage coil (Rapid Biomedical GmbH, Germany) at 9.4T. Shimming was conducted manually using a pulse-acquire sequence giving a linewidth of 47 ± 5 Hz.

Following *ex-vivo* imaging, the brains were then transferred to buffered formal saline before being dispatched for histology.

### 2.5 Quantitative Susceptibility Mapping and T2* mapping

The reconstruction of the *in-vivo* phase data required a pre-processing step to combine the signal from the two receive coils using a global offset correction technique to remove phase shifts between channels (Hammond et al., 2008). *In-vivo* phase data were unwrapped using Laplacian unwrapping (*unwrapLaplacian.m*, MEDI toolbox, Cornell MRI Research Lab, http://weill.cornell.edu/mri/pages/qsm.html (Liu et al., 2011b)) before background field removal using a Variable Sophisticated Harmonic Artifact Reduction for Phase data (VSHARP) (Schweser et al., 2011, Wu et al., 2012) (minimum kernel width = 3 voxels). Thresholded k-space division (TKD) (Shmueli et al., 2009) (threshold t = 5) was then performed on the field map to calculate magnetic susceptibility maps.

*Ex-vivo* QSMs were generated by path-based unwrapping (*unwrapPhase.m,* MEDI toolbox) of the fourth echo of the reconstructed phase data followed by VSHARP background field removal (minimum kernel width = 7 voxels) and TKD (threshold t = 5). QSM using all twelve echoes was attempted by unwrapping the *ex-vivo* phase data temporally and generating field maps using a linear least squares fit at each voxel. However, maps of the R^2^ value from the fitting indicated multiple regions of poor fit which manifested as increased noise and artifacts in the susceptibility maps which were consequently not used for analysis.

Masks used with the VSHARP algorithm were generated automatically as described in the following section. The TKD threshold of t = 5 was chosen to maximise contrast to noise ratio between white matter and grey matter (GM) regions (determined during optimisation of the post processing pipeline).

T2* estimates were calculated for each voxel by fitting a monoexponential signal decay function across all echoes in Matlab.

### 2.6 Registration and mask generation

Regional estimates of magnetic susceptibility and T2* were conducted using a semi-automatic segmentation of the quantitative maps. This required co-registration of the corresponding magnitude images (mean magnitude taken across all echoes for each subject) using an automated software pipeline (Powell, 2017) that incorporated the following steps. Automatic orientation of the brain images by rigid registration to a standard atlas was followed by an intensity non-uniformity correction (N4ITK algorithm) before automatic skull stripping using the STEPS algorithm (Tustison et al., 2010) which also generated masks for each brain. Group-wise registration of echo magnitude images was then performed using NiftyReg software (Modat et al., 2010, Ourselin et al., 2001). First, all subjects were rigidly aligned to a randomly chosen target member of the group. This was followed by 10 iterations of affine registration, producing a matrix describing the global transformations for each brain. There were then 20 iterations of non-rigid registration (NRR). After each iteration, the intensity average image was found from all registered, resampled magnitude images, and used as the target for the subsequent registration. Deformation fields describing the transformation resulting from this non-rigid registration were generated for each brain. These deformation fields were applied to the QSMs and T2* maps of the individual mice to transform them into a common image space. Mean maps of the registered data were then calculated separately for the WT (*in-vivo*: n = 10, *ex-vivo*: n = 8) and rTg4510(*in-vivo*: n = 10, *ex-vivo*: n = 8) groups.

**Fig. 1:**
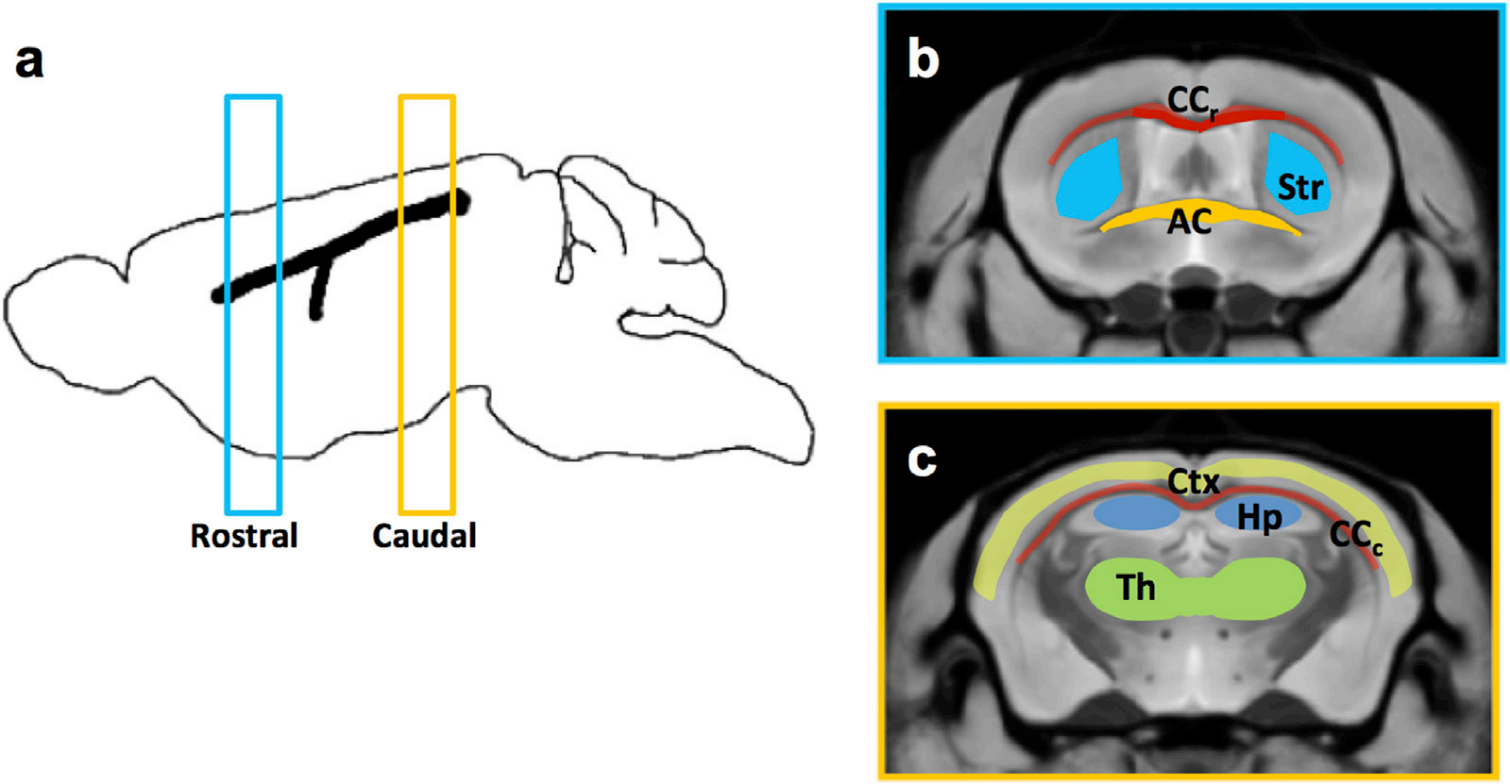
ROIs were drawn in rostral and caudal sections (0.5 mm thickness) in the atlas of the magnitude images(a). In the rostral section(b), ROIs were drawn in the corpus callosum (CC_r_), Striatum (Str) and Anterior Commissure (AC). In the Caudal section (c), regions were drawn in the Cortex (Ctx), the corpus callosum (CC_c_), the Hippocampus (Hp), and the Thalamus (Th)_._

The QSM methodology described here produces magnetic susceptibility measures that are relative rather than absolute (Cheng et al., 2009, Straub et al., 2016). Prior to group comparisons, values at each voxel were recalculated relative to an internal reference region. As reported previously (Argyridis et al., 2014), we used the mean magnetic susceptibility of the whole brain as an internal reference. This was calculated using the brain masks produced by the STEPS algorithm. Selected regions of interested (ROIs) were drawn manually using the mean coregistered average image (Fig. 1) and were propagated back to the quantitative susceptibility and T2* maps, using the deformations from group-wise registration. Mean estimates of quantitative MRI measures were generated by delineation of ROIs in the atlas magnitude images before transformation back into the original image space of each animal where maps were segmented. Slight errors in segmentation propagation, assessed visually, were corrected manually in a small number of regions. ROIs were selected to include WM and GM regions known to be affected by tau pathology at this age (Wells et al., 2015, Sahara et al., 2014). Mean values were calculated in each ROI for comparisons between the rTg4510 and WT groups.

### 2.7 Histology

Brain samples were processed using the Tissue TEK VIP processor (GMI Inc, Ramsey, MN, USA) before being embedded in paraffin wax for coronal brain sectioning (6 μm). Immunohistochemistry (IHC) was performed as previously described (Wells et al., 2015) with antibodies specific for myelin basic protein (MBP, 1:7000; Ab40390, Abcam), phosphorylated tau (PG-5, 1:8000), astrocytes (GFAP, 1:3000; PU020, Biogenex) and microglia (Iba-1, 1:6000; Wako). PG-5 and MBP staining was performed in rTg4510 mice (n = 5) and WT controls (n = 5) at two coronal levels corresponding to the rostral and caudal slices in the MR data (determined manually using the Bregma as an anatomical reference). PG-5, GFAP, Iba-1, and Perls’ Prussian blue (PPB) IHC was carried out in a single selected mouse from each group. IBA-1 and GFAP IHC were performed to identify reactive microglia and astrocytes that are thought to mediate neuroinflammation (Urrutia et al., 2013).

**Fig. 2:**
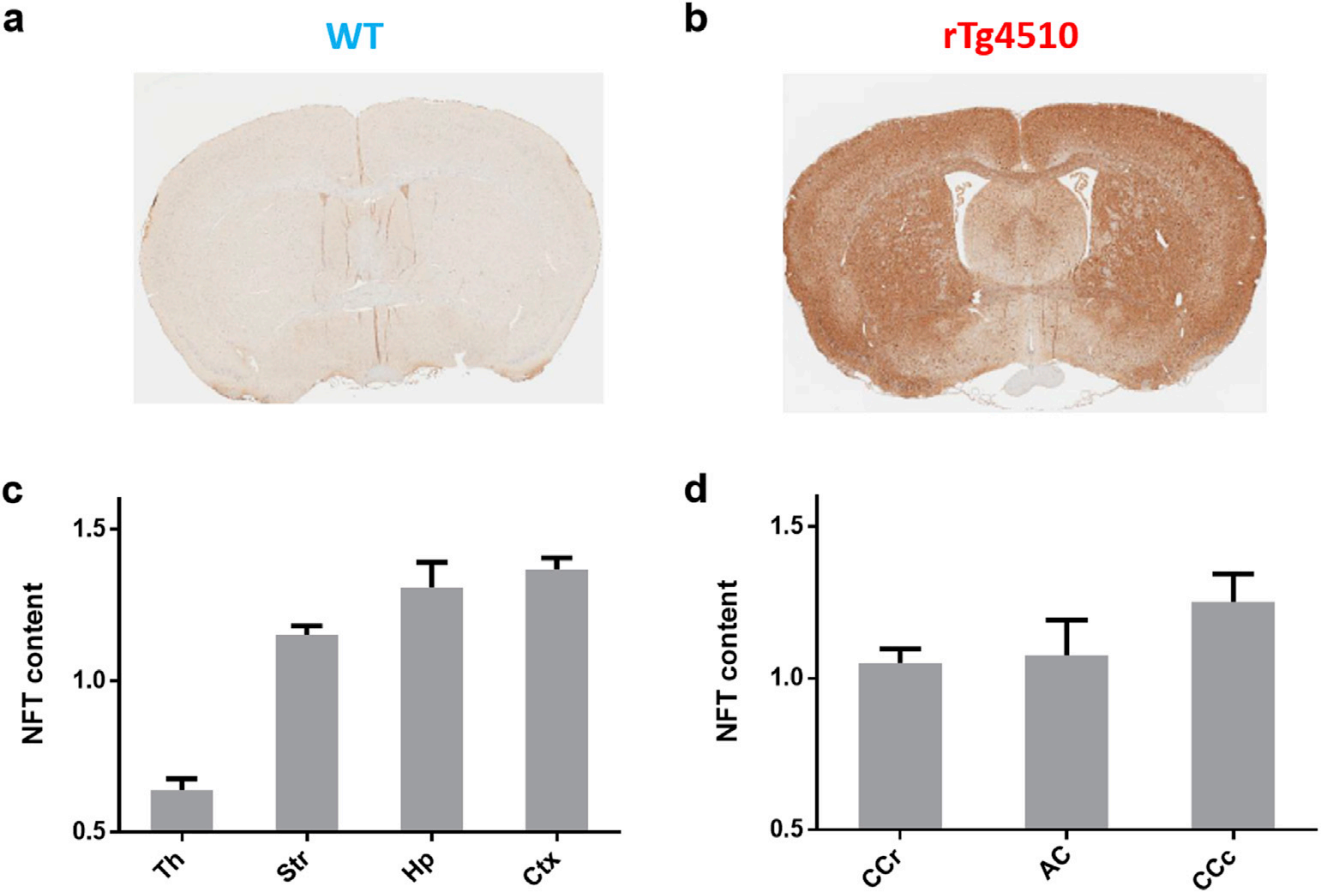
PG-5 staining of NFTs in rostral slice of representative WT(a) and rTg4510(b) mice. NFT content measurements in PG-5 histology are provided in grey matter regions (c) and white matter regions(d) (mean and standard deviation).

Regions of positive PG-5 and MBP staining caused reduced intensity in the digital images of the histology slices. ROIs were segmented manually (ImageJ) in these images and measures of NFT and myelin content in the selected ROIs were produced by subtracting the mean intensity in these regions from the maximum possible greyscale intensity (255) to calculate the intensity reduction caused by PG-5 and MBP staining. It was intended that Tau IHC be used to identify a ‘pathology-free’ control region in rTg4510 tissue that would be used to normalise NFT and myelin content measures to correct for systemic variability in the staining method. However, a pathology free area was not evident in the rTg4510 Tau IHC (Fig. 2b) and consequently a content measure in an ROI drawn over the whole brain tissue region in the image was used for normalisation of the NFT and Myelin measures.

To investigate white matter atrophy, the thickness of the rostral and caudal corpus callosum and anterior commissure were measured. A pixel measurement was taken in the MBP histology images (ImageJ) at a central point in the white matter structure (example location for rostral corpus callosum shown by yellow arrows Fig. 7 a, b).

### 2.8 Statistics

All statistical analysis was performed using Graphpad Prism software (Version 6.01) (Motulsky, 1999). To investigate the relationship between *in-vivo* magnetic susceptibility and T2* estimates with corresponding measurements in high resolution *ex-vivo* data, intra regional comparisons were carried out in the wild-type group using individual t-tests (false discovery rate corrected for multiple comparisons (q ≤ 0.05)). To evaluate inter regional differences in susceptibility, T2*, NFT and MBP intensity, one-way ANOVA comparisons were carried out. The relationship between the regional myelin content and corresponding susceptibility and T2* values in all regions was investigated using Pearson correlations. Finally, to investigate differences between the rTg4510 and WT groups, the mean regional susceptibility, T2* and myelin content were compared between rTg4510 and WT groups with p-values reported from standard two tailed t-tests.

## 3 Results

### 3.1 Regional measurements of NFT content

There was very little PG-5 staining visually apparent in the WT mice compared to the dense and widespread staining in the rTg4510 mice (Fig. 2 a, b). Within the rTg4510 group, all regional measures were significantly different in the grey matter regions (Fig. 2c) (p < 0.05) with the exception of the Hippocampus and Cortex. In agreement with previous studies at this time point (Wells et al., 2015, Holmes et al., 2016), the NFT content was lowest in the thalamus and highest in the cortex with intermediate levels in the striatum and hippocampus.

In white matter regions of the rTg4510, NFT content levels were similar to intermediate levels measured in the grey matter. The NFT content in the rostral corpus callosum was found to be significantly lower than that of the caudal corpus callosum measurement (p < 0.05).

The magnetic susceptibility and T2* measurements for all GM and WM regions were plotted against NFT content estimates from histology in the rTg4510 mice with no significant correlations between parameters observed.

### 3.2 Comparison of mean regional magnetic susceptibility and T2* between WT and rTg4510 mice

**Fig. 3:**
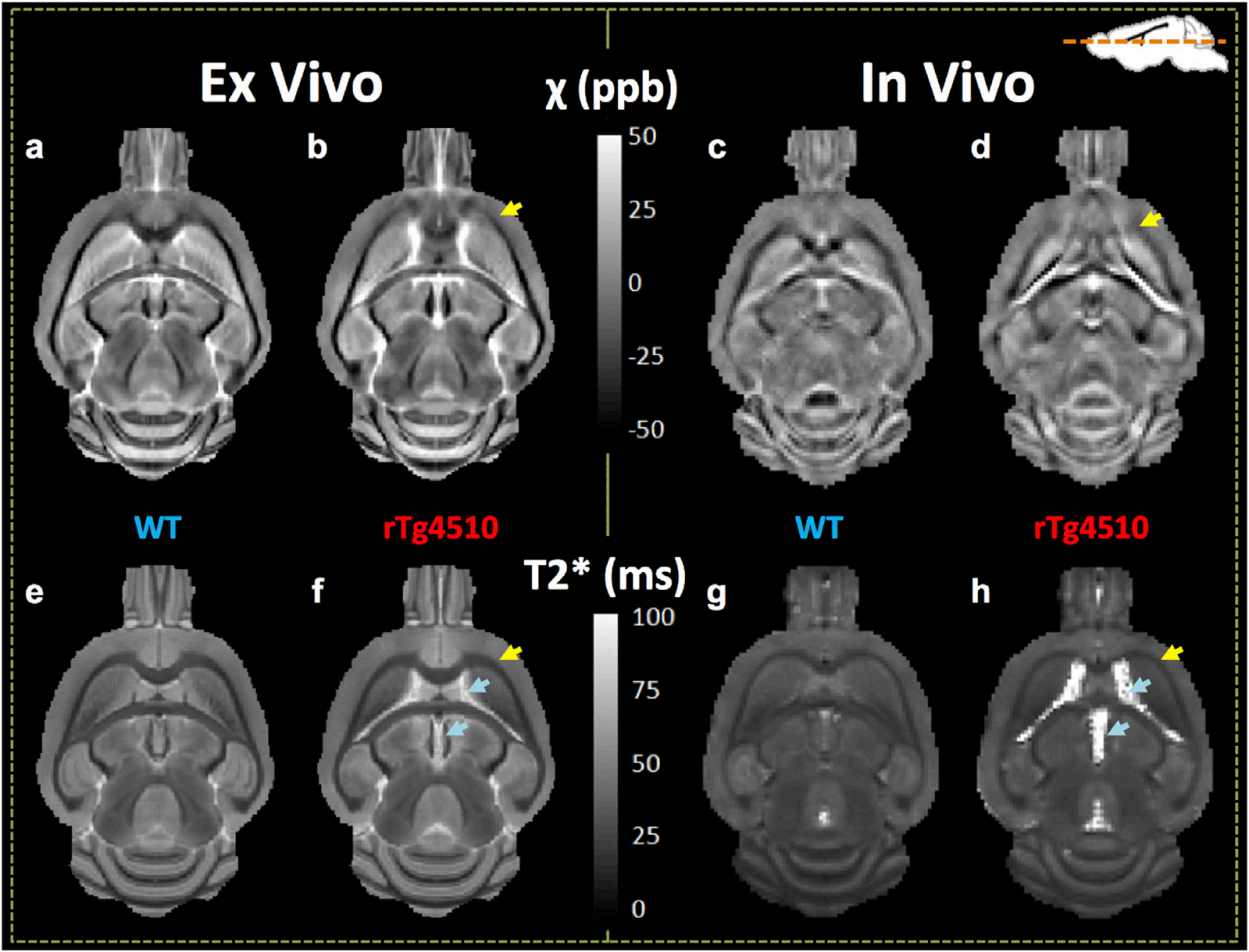
Mean QSM (a–d) and T2*(e–h) images for registered *ex-vivo* (a, b, e, f) and *in-vivo* (c, d, g, h) datasets. A reduction in the grey/white matter contrast can be seen in a rostral section of the corpus callosum in the rTg4510 QSMs that is not apparent in the T2* maps (yellow arrows). Enlarged ventricles are evident in the rTg4510 maps (blue arrows).

An axial slice of mean QSM and T2* maps for each of the registered *in-vivo* and *ex-vivo* datasets is shown in Fig. 3. The anatomical structures were more clearly delineated in the *ex-vivo* data, owing in part to its finer resolution compared to the *in-vivo* maps. Enlargement of the ventricles in the rTg4510 mice is evident in both the QSM and the T2* maps (Fig. 3: Blue arrows in the rTg4510 T2* maps) and has been reported previously (Wells et al., 2015, Holmes et al., 2016). Consistency between *in-vivo* and *ex-vivo* WM-GM contrast is visually apparent in both QSM and T2* maps. Relative to the surrounding grey matter, the white matter regions appeared hypointense indicating a more diamagnetic (negative) susceptibility and shorter T2*. *In-vivo* and *ex-vivo* measurements of magnetic susceptibility and T2* in rTg5410 and WT mice are presented in Table 2. Differences in magnetic susceptibility were detected both *in vivo* and *ex vivo* in the striatum, hippocampus, thalamus, and rostral corpus callosum. In contrast to these regions of low and intermediate NFT content, there were no differences in magnetic susceptibility detected in the cortex, the region of highest NFT burden.

**Table 2:** List of mean and standard deviation of regional measures of magnetic susceptibility and T2*. Abbreviations for white matter regions: CCr – rostral corpus callosum, AC - anterior commissure, CCc caudal corpus callosum. Symbols used in plots: *p < 0.05 **p < 0.01, ***p < 0.001, ****p < 0.0001 denote statistical differences between WT and rTg4510 regional measurements; ^†^p < 0.05 ^††^p < 0.01, ^†††^p < 0.001, ^††††^p < 0.0001 denote statistical differences in WT regional measurements between *in-vivo* and *ex-vivo* data.

ROI	Ex vivo		In vivo	
	WT	rTg4510	WT	rTg4510
χ (ppb)				
Striatum	0.61 ± 0.81	8.71 ± 3.21***	0.54 ± 1.31	4.85 ± 3.09**
Hippocampus	9.45 ± 2.55^††^	13.60 ± 2.91**	5.82 ± 1.86	11.11 ± 4.84**
Cortex	7.01 ± 0.92^††^	2.72 ± 5.37	3.46 ± 3.06	1.40 ± 1.79
Thalamus	1.30 ± 0.97	3.03 ± 1.31**	1.33 ± 1.09	−0.45 ± 1.2**
*CCr*	−34.92 ± 4.29^††††^	−18.73 ± 7.6***	−20.55 ± 6.15	−11.22 ± 5.21**
*AC*	−32.32 ± 1.88^††††^	−31.58 ± 3.33	−13.33 ± 3.45	−9.42 ± 5.34
*CCc*	−46.01 ± 3.97^††††^	−48.70 ± 8.88	−18.31 ± 10.14	−17.10 ± 4.43
T2* (ms)				
Striatum	39.79 ± 1.14^††††^	35.08 ± 1.61****	34.99 ± 1.82	31.58 ± 1.42***
Hippocampus	50.16 ± 1.51^††††^	50.39 ± 2.39	36.89 ± 2.48	36.62 ± 2.55
Cortex	46.14 ± 1.44^††††^	45.13 ± 1.81	33.67 ± 1.4	32.01 ± 1.8*
Thalamus	37.41 ± 1.1^††††^	35.57 ± 1**	31.25 ± 2.44	31.72 ± 0.59
*CCr*	25.23 ± 0.86^††††^	26.67 ± 2.1	22.58 ± 0.82	24.20 ± 3.2
*AC*	24.60 ± 0.73	22.89 ± 0.81***	26.46 ± 2.35	25.27 ± 2.08
*CCc*	24.08 ± 1.07	23.54 ± 1.25	25.02 ± 1.54	24.73 ± 1.72

**Fig. 4:**
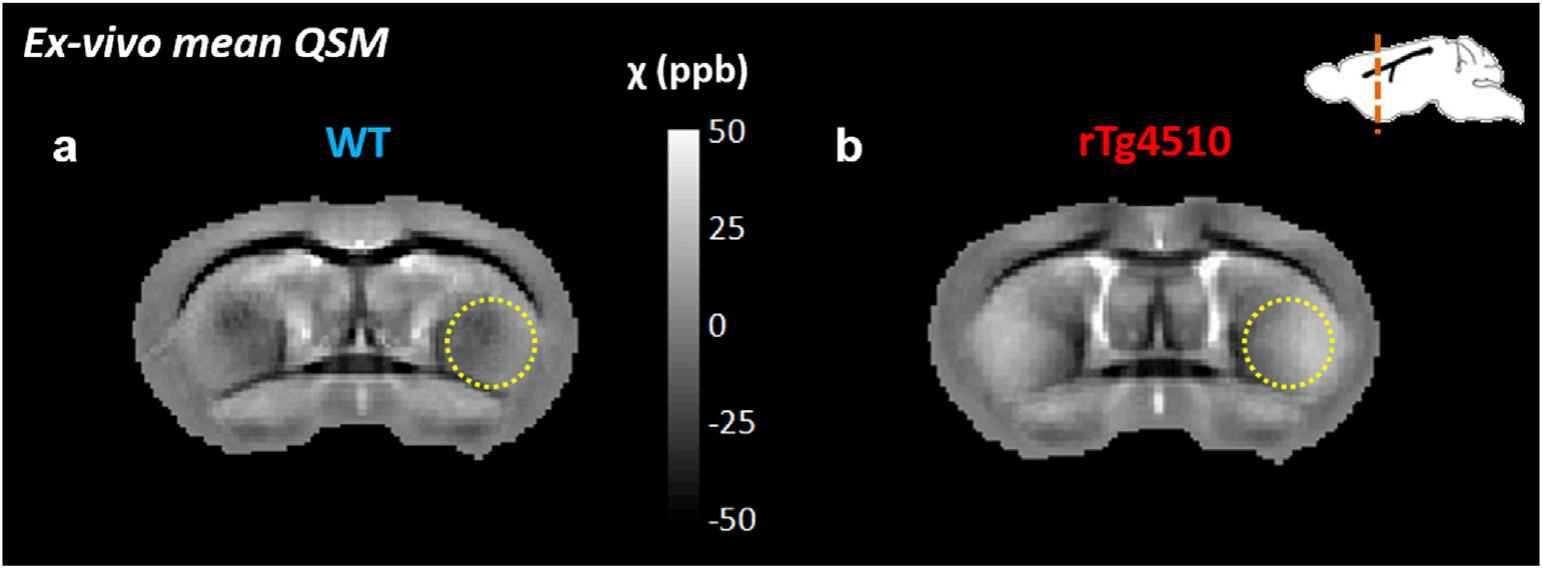
Increased magnetic susceptibility in the striatum of the rTg4510 (relative to WT) was observed visually in group mean QSMs (a, b (yellow circles)).

In grey matter regions, differences in magnetic susceptibility and T2* were most significant in the striatum of the rTg4510. In both the *in-vivo*, and *ex-vivo* datasets, the rTg4510 group had increased paramagnetic susceptibility(p < 0.01) (Table 2) and a reduced T2* (p < 0.001) in the striatum relative to WT controls. This region appears brighter in the *ex-vivo* mean susceptibility maps (yellow circle, Fig. 4 a, b) in the rTg4510. In the hippocampus, the rTg4510 mice had a significantly greater mean magnetic susceptibility than the WT mice both *in vivo* (p < 0.01) and *ex vivo* (p < 0.01), with no significant differences in T2* (Table 2). In the cortex, a significant shortening of T2* in the rTg4510s was observed *in vivo* only (p < 0.05). The mean susceptibility *ex vivo* was significantly elevated in the rTg4510 thalamus which also had a significantly reduced T2*(p < 0.01), findings that were not replicated *in vivo* where magnetic susceptibility was found to be significantly decreased (p < 0.01) with no significant change in T2*.

In addition to the susceptibility differences in the rostral corpus callosum of the rTg4510 mice, an *ex-vivo* reduction in T2* (p < 0.001) was observed in the anterior commissure white matter region. In the caudal corpus callosum, the WM region exhibiting the highest NFT content, no differences in MRI measurements were detected in the rTg4510 mice.

Focusing on the WT data (Table 2), we observed regional differences in the magnetic susceptibility and T2* between the *in-vivo* and *ex-vivo* data, presumably due to *ex-vivo* tissue processing. Broadly, magnetic susceptibility and T2* increased in *ex-vivo* GM tissue regions (Table 2, p ≤ 0.01) (with the exception of susceptibility in the Thalamus and Striatum). In WM regions, magnetic susceptibility was decreased in the *ex-vivo* tissue (Table 2, p ≤ 0.0001) with no differences observed in T2* (with the exception of the rostral corpus callosum).

### 3.3 Regional measurements of myelin and iron content

**Fig. 5:**
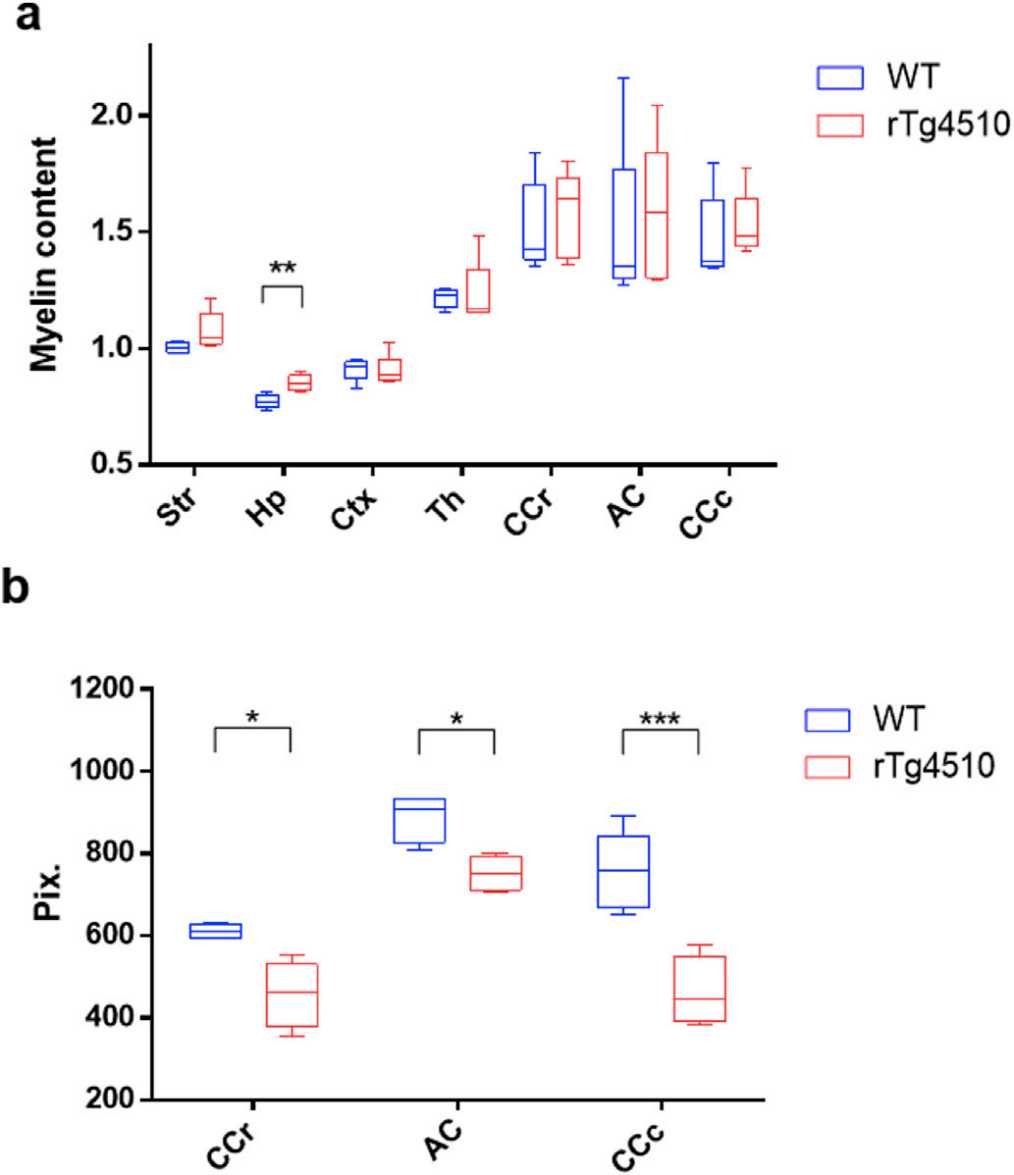
Myelin content (a) and white matter structure thickness measurements(b) in WT and rTg4510 mice (n = 5). White matter thickness was measured by number of pixels in the histology images. Symbols used in plots: *p < 0.05 **p < 0.01, ***p < 0.001.

Differences in myelin content between the rTg4510 and WT mice were evaluated through comparison of the regional estimates from the IHC analysis. The hippocampus was the only region to exhibit a difference with myelin content increased in the rTg4510 group relative to the WT group (p < 0.01) (Fig. 5a). A reduction in the thickness of white matter structures was visually apparent in the rTg4510 compared to WT mice. Measurements of tract thickness were taken in the MBP histology images (Fig. 5b) and a significant reduction in was identified in all white matter structures (p < 0.05). The decrease was most significant in the caudal corpus callosum (P < 0.001). The percentage decrease in the mean thickness of WM structures in the rTg4510 mice relative to the WTs were calculated as 39% for the caudal corpus callosum, 25% for the rostral corpus callosum, and 25% for the anterior commissure.

There were no visually apparent regions of positive PPB staining evident in the selected WT and rTg4510 mice (Supplementary Fig. S1).

### 3.4 Correlation of myelin content measurements with MRI parameters

**Fig. 6:**
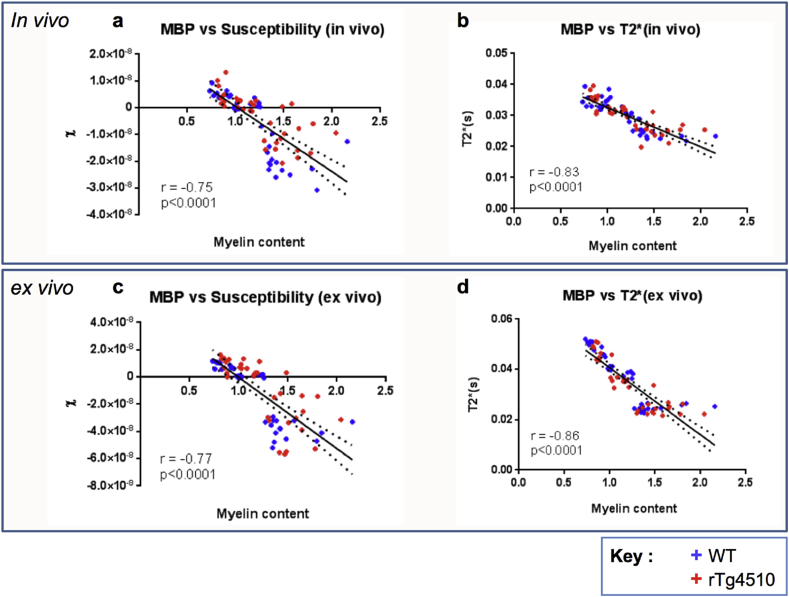
Regional myelin content (MBP intensity reduction) vs *in-vivo* (a, b) and *ex-vivo* (c, d) magnetic susceptibility (a, c) and T2*(b, d) measurements in all brain regions (n = 7) for both WT(n = 5) and rTg4510 (n = 5) mice. Pearson's coefficient r (given to two decimal places) and p values for correlations are provided. The best-fit line is shown with dotted lines indicating the 95% confidence band.

The magnetic susceptibility and T2* measurements for all GM and WM regions were plotted against myelin content estimates from MBP histology. Significant correlations were identified between myelin content measurements and magnetic susceptibility (p < 0.0001, for *in-vivo* and *ex-vivo* data) and T2* (p < 0.0001, for *in-vivo* and *ex-vivo* data) when animals from both rTg4510 and WT cohorts (n = 5 for each) were included in the analysis (Fig. 6). Significant correlations were also observed between these variables when analysing WT and rTg4510 groups separately (p < 0.0001). These correlations indicated a relationship between increasing myelin content and decreasing magnetic susceptibility and T2* in the brain regions sampled.

**Fig. 7:**
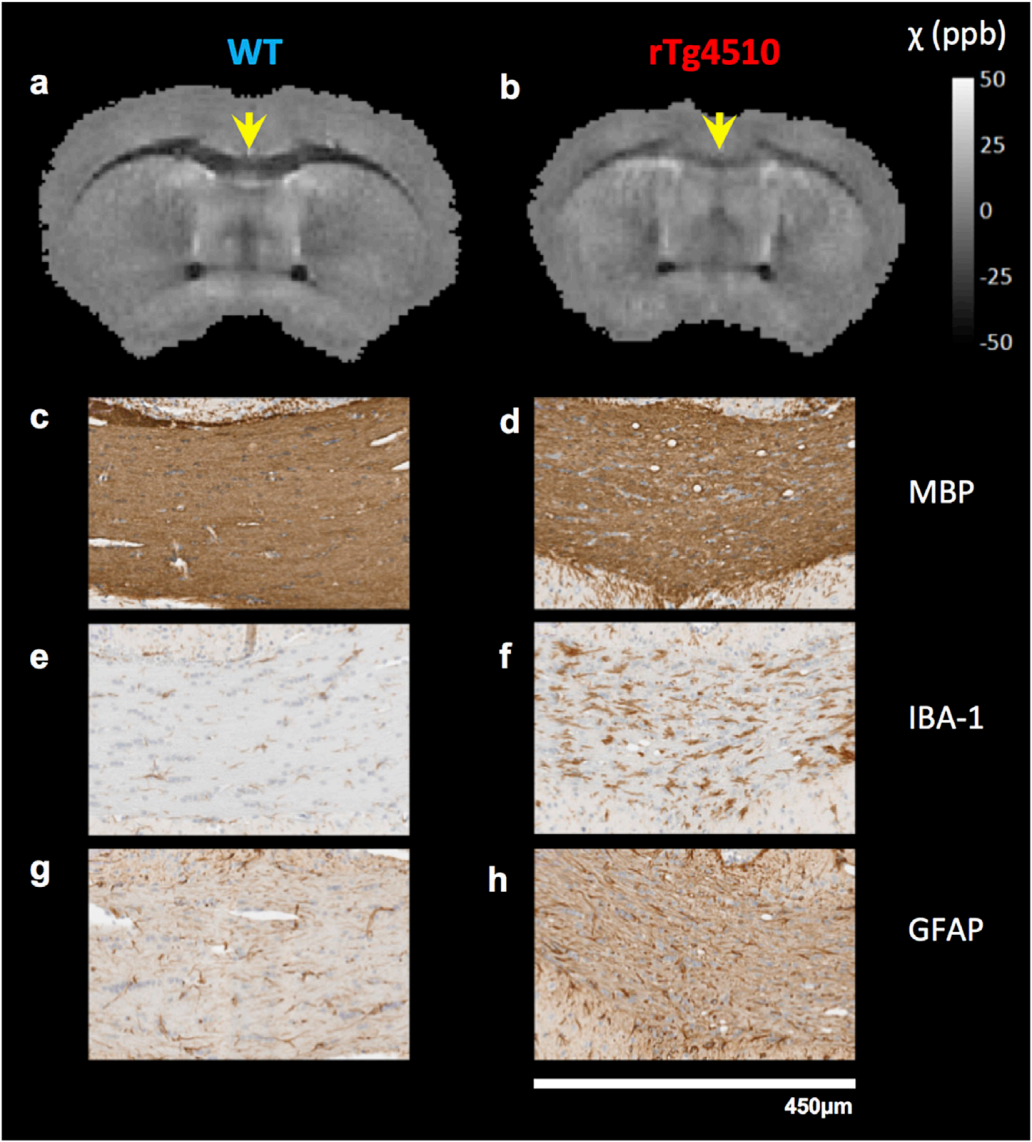
QSMs and histology in the rostral section of the corpus callosum in representative mice(a-h). A reduction in grey matter/white matter contrast was apparent in rTg4510 *ex vivo* QSMs (a, b). Staining in the genu section of the corpus callosum (yellow arrows) showed rarefaction and vacuolisation in myelin (c, d), and increased microglial (e, f) and astrocytic (g, h) activity.

### 3.5 Markers of neuroinflammation in regions of intermediate and low NFT deposition

In the genu of the corpus callosum, findings of increased magnetic susceptibility were accompanied by a disorganised arrangement of myelin in the rTg4510, with rarefaction and vacuolisation evident in MBP staining (Fig. 7c and d). An increased density of staining for reactive microglia (Fig. 7e and f) and astrocytes (Fig. 7g and h) was also observed in the rTg4510 relative to WT controls. Increased GFAP and IBA-1 staining were also visually apparent in other regions of intermediate and high NFT content (not shown).

**Fig. 8:**
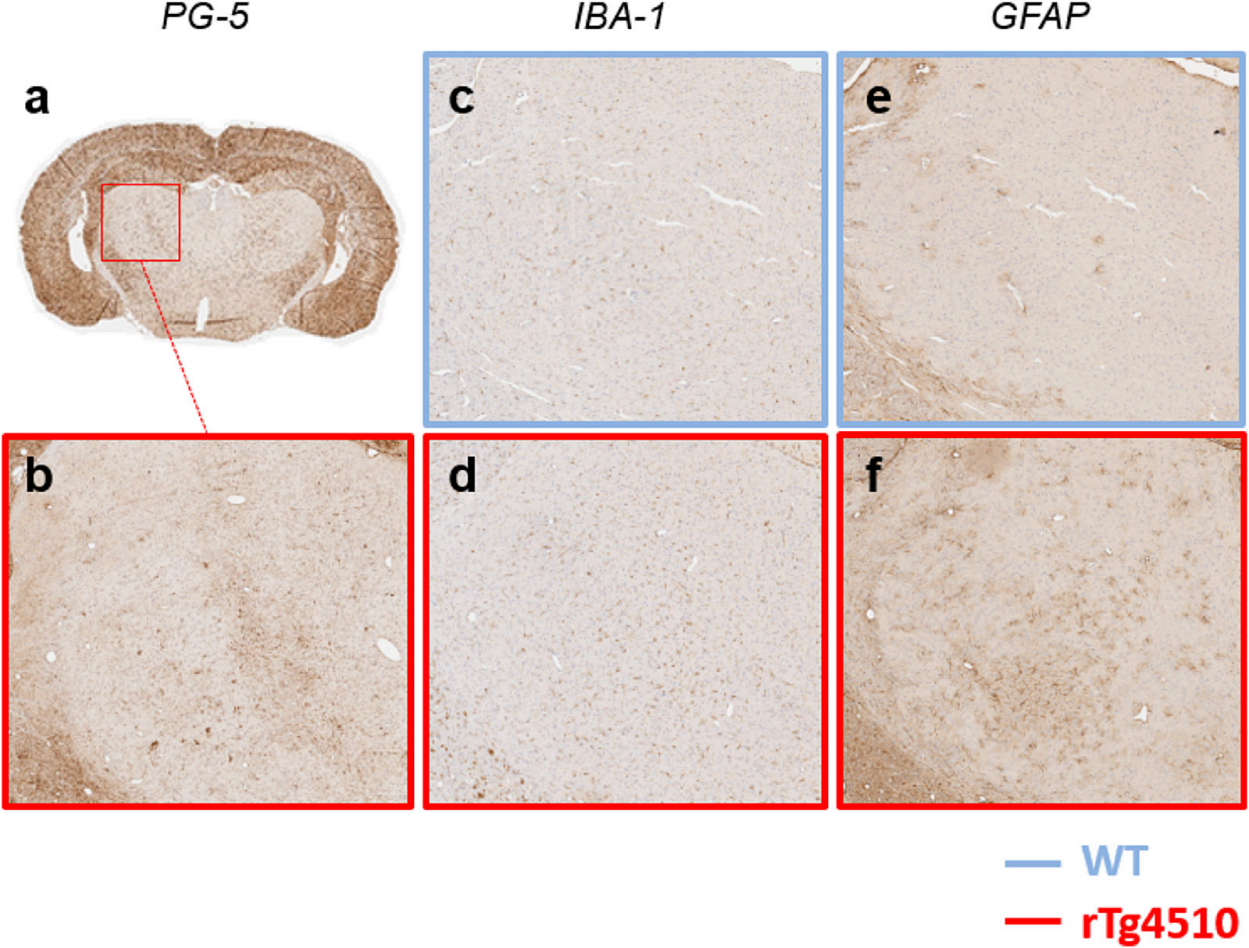
PG-5 staining in a region of low NFT deposition (a, b) in the thalamus (rTg4510), and IBA-1 (c, d) and GFAP (e, f) staining in the same region in a WT (c, e) and rTg4510 (d, f) mouse.

In the Thalamus, a region of low NFT deposition, increased levels of IBA-1 (Fig. 8c and d) and GFAP (Fig. 8e and f) staining were visually apparent in a region of minimal NFT staining (Fig. 8b).

## 4 Discussion

In this study we have used semi-automated segmentation techniques to provide a comparison of magnetic susceptibility and T2* measures in selected regions in the rTg4510 mouse model of tauopathy with values in WT controls. This mouse model accumulates NFTs throughout the forebrain progressively with age in a manner similar to the accumulation observed in the human form of AD. To support *in-vivo* QSM findings, high resolution imaging and myelin sensitive staining were performed on *ex-vivo* tissue samples from the same cohort of mice. Differences in magnetic susceptibility were observed in regions of low and intermediate NFT burden along with an increased presence of markers of neuroinflammation. Correlations between MRI and MBP measures indicate that myelin may be a key driver of regional susceptibility and T2* values. These findings suggest that tissue magnetic susceptibility provided by QSM may be useful as a non-invasive marker of tau pathology in its early stages.

### 4.1 Ex-vivo vs. in-vivo comparisons in WT mice

Unlike clinical applications of the QSM technique, animal imaging offers the additional possibility of comparing susceptibility values *in vivo* with those acquired subsequently in the same group of subjects *ex vivo*. Relative to *ex-vivo* acquisition, additional error may be introduced into *in-vivo* datasets from increased noise and motion artifacts, primarily of physiological origin that fluctuate with the cardiorespiratory cycle (Jack et al., 2005, Oguz et al., 2013). Errors may also arise from partial volume effects due to the lower resolution attainable *in vivo*. Furthermore, the use of 100% O2 to deliver anaesthetic may alter levels of deoxyhemoglobin in the blood, thereby affecting the physiological accuracy of susceptibility estimates. This might suggest that a fairer group comparison can be achieved using *ex-vivo* data. However, tissue fixation processes and the reduction in imaging temperature will alter relaxation properties (Shepherd et al., 2009, Rieke and Pauly, 2008) and susceptibility values (Quesson et al., 2000, Poorter et al., 1995) so that measurements may not reflect true values *in vivo*. To evaluate the effects of these differences, QSM and T2* data were collected both *in vivo* and *ex vivo* in the same cohort of mice. Significant increases in both measures were observed *ex vivo* in grey matter regions in the wild-type mice (Table 2). In the case of T2*, this may have been caused by the fixation and rehydration processes, known to lengthen the T2 (and therefore T2*) properties of the *ex-vivo* tissue (Shepherd et al., 2009) relative to *in-vivo* tissue. Increases in *ex-vivo* susceptibility measurements in the grey matter regions may be due to the known inverse relationship between magnetic susceptibility and temperature of paramagnetic materials, which has been used to adjust *ex-vivo* susceptibility values previously (Langkammer et al., 2012). White matter regions displayed an increased negative susceptibility *ex vivo* than measured *in vivo.* This finding is supported by a previous study in the optic nerve in rats (Luo et al., 2014) but may also be due to partial volume errors in the *in-vivo* analysis. The *in-vivo* voxel size used (150 μm isotropic) was large relative to the white matter structures in the mouse brain and incorporation of grey matter into ROIs may have increased the mean and standard deviation of susceptibility measurements relative to the *ex-vivo* data, which employed smaller voxels (80 μm isotropic).

### 4.2 Susceptibility and T2* differences between regions within mice

Differences in magnetic susceptibility estimates between grey and white matter regions were in good agreement with previously published values for *in-vivo* (Klohs et al., 2011, Klohs et al., 2013) and *ex-vivo* (Liu et al., 2011a) wild-type mice. Both *in-vivo* and *ex-vivo* datasets displayed a pattern of lower values of magnetic susceptibility and T2* in the striatum and thalamus relative to the grey matter regions in the cortex and hippocampus. These reduced values may be due to the greater myelin content measured in the striatum and thalamus. Myelin is diamagnetic and produces a shortening of T2* and reduces the bulk magnetic susceptibility of tissue (Liu et al., 2011a, Klohs et al., 2013, Lee et al., 2012, Dibb et al., 2014). The possibility that myelin plays a key role in the MRI measures in the WT cohort is supported by the correlations between regional susceptibility, T2*, and myelin content measurements. A relationship between decreasing magnetic susceptibility and T2*with increasing myelin content was demonstrated in the *in-vivo* and *ex-vivo* data for both the WT and rTg4510 groups. These results appear to show that myelin concentration in the brain is a major contributor to magnetic susceptibility and T2* measured using MRI and that increasing myelin levels reduce both the magnetic susceptibility and T2* values (Stüber et al., 2014).

### 4.3 Group comparisons between rTg4510 and WT mice

In comparisons of regional susceptibility estimates between the WT and rTg4510 groups, the rTg4510s exhibited differences both *in vivo* and *ex vivo* in the striatum, hippocampus, thalamus and rostral corpus callosum. NFT content measurements indicate that these are regions of low and intermediate tau pathology. Regions of highest NFT content measures in the grey matter(cortex) and white matter (caudal corpus callosum) (see Fig. 2) did not exhibit susceptibility differences which may be due to the advanced stage of the pathology. Relative to other WM regions, elevated NFT levels in the caudal corpus callosum were accompanied by a larger and more significant reduction in structural thickness measured in the rTg4510 (Fig. 5b) which suggest a greater level of neurodegeneration has occurred. In the cortex, the region of greatest pathology in grey matter regions, severe atrophy at this time point has been reported previously (Wells et al., 2015, Holmes et al., 2016). Furthermore, NFT formation has been detected in the cortex at the earliest stages of pathology in the rTg4510 (Ramsden et al., 2005b).

A decreased diamagnetism in the rostral corpus callosum was detected both *in vivo* and *ex vivo* in the rTg4510 (Table 2) and a loss of WM-GM contrast was evident in the mean susceptibility maps (Fig. 3). Observations of vacuolisation and rarefaction in MBP staining in this region (Fig. 7) were consistent with a previous study in which electron microscopy observations identified myelin abnormalities from as early as 4 months in the rTg4510 mouse (Sahara et al., 2014). The increased GFAP and IBA-1 staining in this region respectively indicate increased reactive microglia and astrocytes associated with neuroinflammation. These processes have previously been observed to occur as a result of demyelination in AD (Akiyama et al., 2000). However, there was no difference in myelin content measurements relative to WT mice suggesting that at this time-point, demyelination in the rTg4510 is not the root cause of susceptibility changes.

With the exception of the hippocampus, no significant differences were detected in the regional mean myelin content measurements between the rTg4510 and WT groups. Greater levels were observed in the hippocampus of the rTg4510 which may reflect increased myelin. Stress has previously been implicated as a cause of increased hippocampal myelin (Chetty et al., 2014). However, this finding is contrary to the QSM results which showed an increase in hippocampal susceptibility in the rTg4510s. It may be possible that the increased myelin content measurement is a false positive, caused by a limitation in the staining measurements protocol. In the slices selected, NFTs were prevalent throughout the rTg4510 tissue so there was no obvious ‘pathology free’ region that could be used as a control to normalise for variability in PG-5 and MBP stains applied. Consequently, the mean of the stain intensity across the whole brain region was used as a reference. While this should be satisfactory for the intra-group correlation results, inherent pathology-driven myelin differences in the whole brain reference in the rTg4510 mice may introduce error into inter group comparisons. In both the rostral and caudal slices, a slight decrease in the myelin content measure in the whole brain reference region was observed in the rTg4510 relative to the WT (this increase was not statistically significant).

Iron accumulation is another known cause of paramagnetic increases in tissue in AD (Connor et al., 1992, Parsey and Krishnan, 1998) and has previously been associated with tau pathology and inflammatory responses. Iron has been identified in NFTs (Good et al., 1992) that form in the AD brain and can also give rise to increases in magnetic susceptibility estimates and T2* shortening (Langkammer et al., 2012, Acosta-Cabronero et al., 2013, Stüber et al., 2014). The results of PPB staining for iron, a stain used previously to identify iron deposition in AD mouse models (El Tannir El Tayara et al., 2006, Jack et al., 2004), were negative in the rTg4510 mouse (Supplementary Fig. S1). However, DAB intensification of PPB IHC has been used in previous studies to provide enhanced sensitivity to iron in the mouse brain (Lee et al., 2004, Wengenack et al., 2011) and could be applied to more conclusively exclude the possibility of excess iron accumulation in the rTg4510.

In the thalamus, low levels of tau pathology were detected in the rTg4510 mice, and has been observed previously that structural volume loss does not occur at this time point (Wells et al., 2015). Neuroinflammatory processes are thought to precede and cause neurodegeneration in Alzheimer's Disease (Morales et al., 2014) and may contribute to greater blood brain barrier permeability (Zenaro et al., 2016). Reported increases in MR diffusion and perfusion measurements support the possibility of blood barrier breakdown in the thalamus of the rTg4510 (Wells et al., 2015). This would lead to an alteration in composition of the interstitial space, altering levels of water and macromolecules which will cause changes in the tissue bulk magnetic susceptibility (Quarantelli, 2015). With the progression of tau pathology, neurodegeneration has been shown to occur in the rTg4510. This will cause a loss of cells, axons, and myelin, and the tissue causing bulk magnetic susceptibility to be altered in a manner that opposes early stage changes (Zhao et al., 2016, Zhao et al., 2017) and may explain the lack of differences detected in regions of greatest NFT burden. The balance of competing effects of tau pathology and neurodegenerative processes on bulk magnetic susceptibility will differ between brain regions in the rTg4510 mice at the time point imaged, and may be responsible for the lack of an observed correlation between QSM and NFT density parameters. Further work would be required to unpick the biological alterations that occur during neuroinflammatory and neurodegenerative processes and their effects on tissue magnetic susceptibility.

The QSM and T2* techniques presented here can be acquired simultaneously using a single pulse sequence and the differing mechanisms that drive their contrast suggest that they may offer complementary information (Shmueli et al., 2009, Deistung et al., 2013). There were a small number of regions where group differences were identified in both the susceptibility and T2* maps. An increased magnetic susceptibility and decreased T2* was observed in the rTg4510 relative to wild-type controls *in vivo* and *ex vivo* in the striatum and *ex vivo* only in the thalamus. In the rostral corpus callosum and hippocampus, *in-vivo* and *ex-vivo* increases in magnetic susceptibility were observed in the rTg4510 in the absence of T2* changes. This may reflect greater sensitivity of magnetic susceptibility to changes in the rTg4510. Magnetic field inhomogeneities arising from background gradients can cause reductions in T2* estimates and may introduce a bias into group comparisons. Maps of field inhomogeneity (ΔB_0_) were generated to investigate effects of these contributions and no differences were identified between WT and rTg4510 datasets *in vivo* or *ex vivo* (Supplementary Fig. S2).

T2* displayed a correlation with myelin content measures that was of greater statistical significance than that for QSM. Possible sources of increased variability in the susceptibility estimates may have arisen through normalisation to a reference region and the vulnerability of this QSM protocol to magnetic susceptibility anisotropy effects. Reference regions are commonly used in QSM studies (Bilgic et al., 2012, Acosta-Cabronero et al., 2013, Straub et al., 2016) and may introduce error through a lack of consistency between subjects. However, it has been found previously that effects of normalisation are minimal relative to measureable differences in magnetic susceptibility (Acosta-Cabronero et al., 2016). To reduce the effects of susceptibility anisotropy on measurement comparisons, *in-vivo* and *ex-vivo* brains were positioned and secured as consistently as possible between mice. However, there is likely to be some variation in the orientation of brains relative to the B0 field and future work could use susceptibility tensor imaging (Liu, 2010) to investigate these effects.

T2* mapping was conducted using multi-echo data, in contrast to the single-echo flow-compensated sequence that was used to acquire data for QSM. The use of multi-echo data to generate field maps using recently developed phase unwrapping techniques such as CAMPUS (Feng et al., 2013) or non-linear field map estimation (Liu et al., 2013) may reduce noise and improve magnetic susceptibility estimates (Emma Biondetti and Shmueli, 2016). Further work to modify the multi-echo sequence to include first order gradient moment nulling for flow compensation at every echo time (Xu et al., 2014, Wu et al., 2016) would enable simultaneous *in-vivo* acquisition of data for both T2* and susceptibility mapping. However, it may not be necessary to use flow-compensation at 9.4 T, especially when comparing regions which do not contain large vessels. Further analysis including calculation of susceptibility maps from the multi-echo T2* data acquired *in vivo* in this study and a comparison of the resulting susceptibility values with the single-echo (Sun and Wilman, 2015, Biondetti et al., 2017) values would shed light on this. Scan time could be further reduced using an Echo Planar readout for inclusion in an *in-vivo* multi-parametric AD imaging protocol (Mesrob et al., 2012, Walhovd et al., 2009, Cherubini et al., 2010, Yang et al., 2011, Lane et al., 2017).

## 5 Conclusion

In this study, T2* mapping and QSM were conducted in a mouse model of AD exhibiting tau pathology for the first time. The results indicate that both techniques are sensitive to regional differences in myelin content in the mouse brain *in vivo.* Magnetic susceptibility differences were observed in regions that exhibited low NFT burden and increased staining for reactive microglia and astrocytes. QSM in the rTg4510 may therefore constitute a novel *in-vivo* biomarker of neuroinflammation in the rTg4510 and a means by which to detect early tau pathology and to test therapeutics. The QSM protocol implemented in this work is analogous to clinical protocols that are currently available and therefore could be simply incorporated into studies of human Alzheimer's Disease and other tauopathies.
