# Supplementary material for: Tissue magnetic susceptibility mapping as a marker of tau pathology in Alzheimer's disease
Source: Neuroimage. 2017 Oct 1;159:334–45. doi: 10.1016/j.neuroimage.2017.08.003 (PMC5678288; doi:10.1016/j.neuroimage.2017.08.003)
Supplement: Supplementary file 1 [file mmc1.doc]

## Supplementary materials


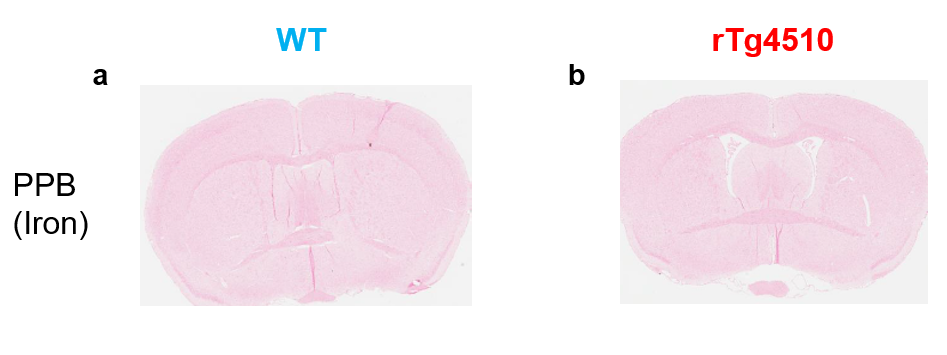


**S 1.** PPB staining in a rostral slice of a representative wild type control (a) and rTg4510 (b).


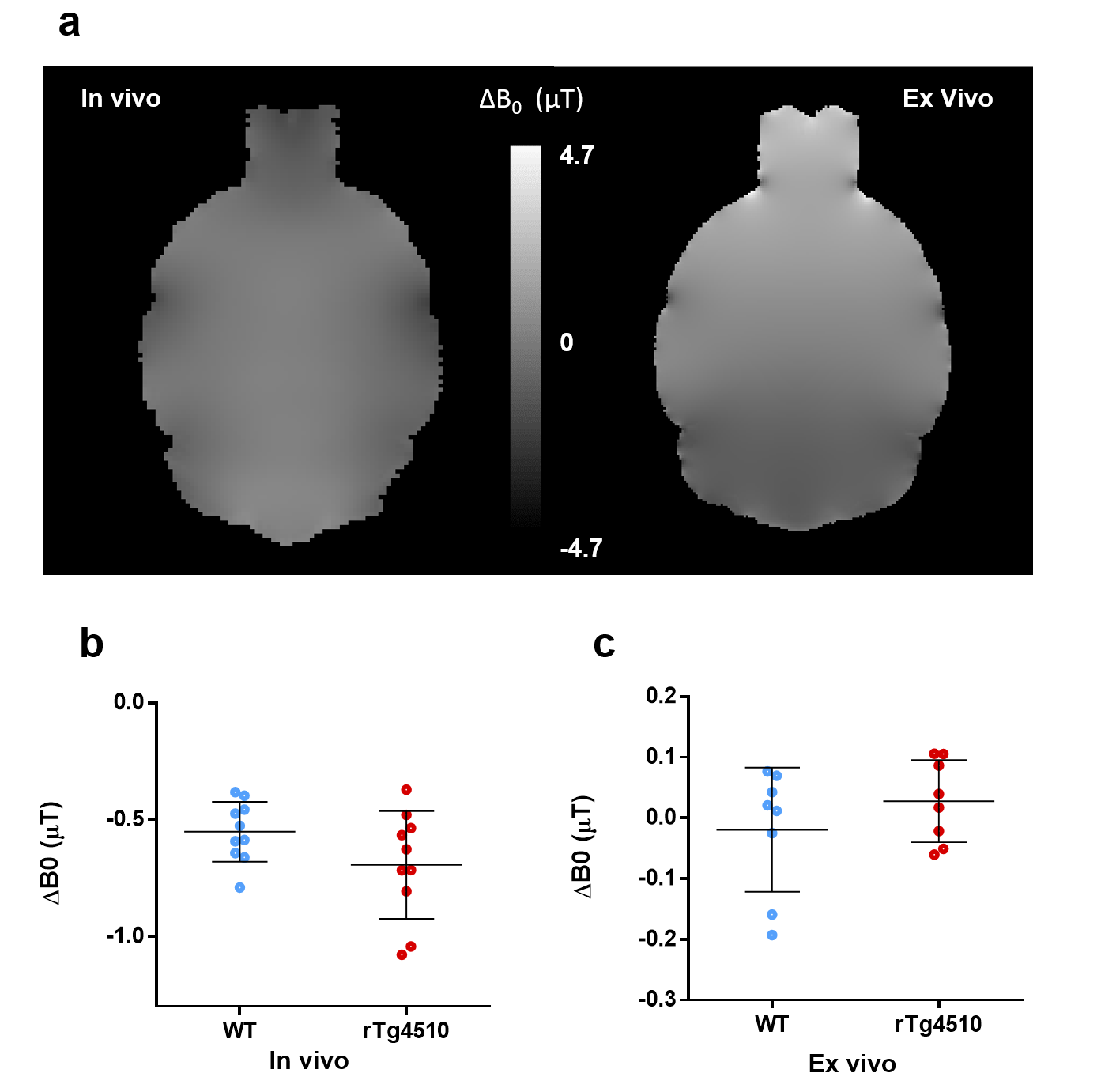


S 2. Examples of *in-vivo* and *ex-vivo* background magnetic field maps (a), and comparison of mean ∆B_0_ map values within in the brain *in vivo*(b) and *ex vivo*(c) in WT and rTg4510 mice. Field maps (a, b) were generated by removal of local field components (used for QSM) from the unwrapped phase data using VSHARP. There were no significant differences in mean absolute ∆B_0_ values measured over the whole brain between WT and rTg4510 mice *in vivo* (fig b, p=0.1, two tailed t-test) or *ex vivo* (fig c, p=0.3, two tailed t-test).
